# How Covid-19 changed psychiatric department admissions: observational study comparison of psychiatric department admissions over three years

**DOI:** 10.1192/j.eurpsy.2023.979

**Published:** 2023-07-19

**Authors:** T. Barlattani, C. D’Amelio, A. Russo, R. Santini, F. Pacitti, A. Rossi

**Affiliations:** 1 Department of Biotechnological and Applied Clinical Sciences, University of L’Aquila, L’Aquila; 2 Department of System Medicine, University of Rome Tor Vergata, Rome, Italy

## Abstract

**Introduction:**

Although the role of SARS-CoV2 pandemic on psychiatric Emergency Department (ED) encounters, has been analysed, few studies have focused on the pandemic influence on patients’ characteristics and admission rates in a psychiatric ward.

**Objectives:**

The aim of this cross-sectional study is to analyse characteristics of patients admitted in the psychiatric ward of “San Salvatore” Hospital in L’Aquila (Italy) in a three-year timeframe, from 2019 to 2021, and evaluate the impact of COVID-19 pandemic on admission trends over the course of these three years.

**Methods:**

We collected data regarding 1115 patients from the hospital discharge summaries (Scheda di Dimissione Ospedaliera, SDO) of “San Salvatore” Hospital. Patients were sorted according to diagnosis, year of admission and age range. Comparison of mean values from each group was attained using the Student’s t-test, while percentages and ratios were compared by means of the Chi-Square test.

**Results:**

Between January 2019 and December 2021, 1115 patients were admitted in our psychiatric ward. In 2020, during pandemic outbreak, we observed a reduction of the number of admissions, with 351 patients. Although no statistically significant differences were found regarding patients’ gender or age, we observed a higher number of male patients admitted during all three years (male/female ratio: 231/171 in 2019, 217/134 in 2020 and 192/170 in 2021). Admission rates of patients aged between 18 to 30 years were higher during 2020 and 2021, conversely in the previous year the most represented group were patients aged between 41 to 50 years. Regarding diagnostic categories, percentages remained relatively steady during the three years. Nevertheless, it was possible to observe a slight reduction of Schizo-Psychotic disorders (175/402; 45,53% in 2019, 135/351; 38.46% in 2020 and 119/362; 32,87% in 2021) and a slighter reduction of Personality disorders percentages (49/402; 12,19% in 2019, 37/351; 10,54% in 2020 and 36/362; 9,94% in 2021). Conversely, results showed an increase in admission of patients with substance use disorder: 24 out of 402 patients (5,97%) in 2019, 32 out of 351 patients (9,12%) in 2020, and 46 out of 362 patients (12,71%) in 2021.

**Image 2:**

**Figure:**
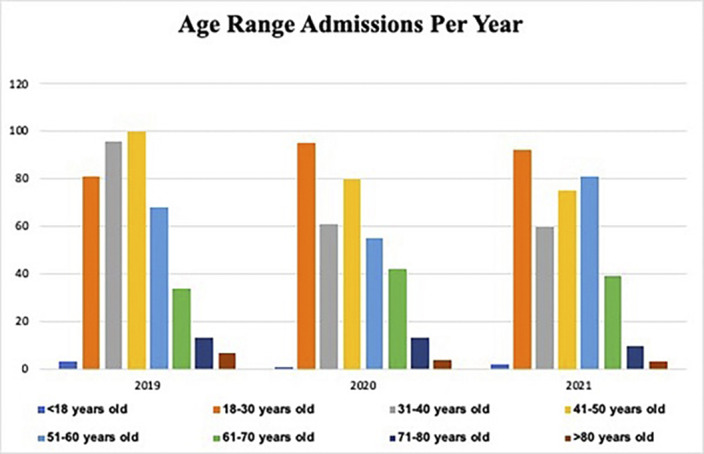

**Image 3:**

**Figure:**
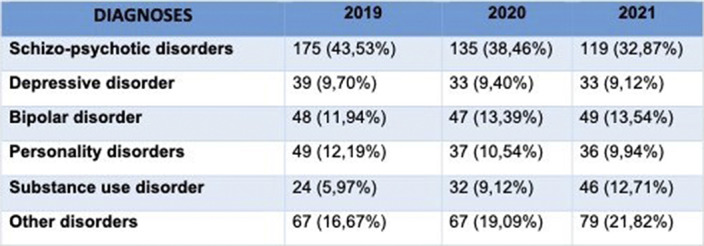

**Conclusions:**

Despite biases due to the one-site evaluation, the strength of the present study relies in the retrospectively cross-sectional observation design conducted to evaluate a three-year timeframe, spanning throughout the pandemic. The sharp reduction of admissions rates in 2020, is in line with other data regarding ED accesses’ trends during pandemic. Increasing rates of admission of patients between 18 and 30 years could be a result of a stronger impact of the pandemic on young people’s mental health. Moreover, increasing trends of admission of patients with substance abuse disorders may be potentially addressed to distress symptoms brought by the pandemic.

**Disclosure of Interest:**

None Declared

